# Reduction of Surgical Site Infections after Implementation of a Bundle of Care

**DOI:** 10.1371/journal.pone.0044599

**Published:** 2012-09-04

**Authors:** Rogier M. P. H. Crolla, Lijckle van der Laan, Eelco J. Veen, Yvonne Hendriks, Caroline van Schendel, Jan Kluytmans

**Affiliations:** 1 Department of surgery, Amphia Hospital, Breda, The Netherlands; 2 Laboratory for Microbiology and Infection Control, Amphia Hospital, Breda, The Netherlands; 3 Operating Theatre, Amphia Hospital, Breda, The Netherlands; 4 Department of Medical Microbiology and Infection Control, VU University Medical Center, Amsterdam, The Netherlands; University of Louisville, United States of America

## Abstract

**Background:**

Surgical Site Infections (SSI) are relatively frequent complications after colorectal surgery and are associated with substantial morbidity and mortality.

**Objective:**

Implementing a bundle of care and measuring the effects on the SSI rate.

**Design:**

Prospective quasi experimental cohort study.

**Methods:**

A prospective surveillance for SSI after colorectal surgery was performed in the Amphia Hospital, Breda, from January 1, 2008 until January 1, 2012. As part of a National patient safety initiative, a bundle of care consisting of 4 elements covering the surgical process was introduced in 2009. The elements of the bundle were perioperative antibiotic prophylaxis, hair removal before surgery, perioperative normothermia and discipline in the operating room. Bundle compliance was measured every 3 months in a random sample of surgical procedures.

**Results:**

Bundle compliance improved significantly from an average of 10% in 2009 to 60% in 2011. 1537 colorectal procedures were performed during the study period and 300 SSI (19.5%) occurred. SSI were associated with a prolonged length of stay (mean additional length of stay 18 days) and a significantly higher 6 months mortality (Adjusted OR: 2.71, 95% confidence interval 1.76–4.18). Logistic regression showed a significant decrease of the SSI rate that paralleled the introduction of the bundle. The adjusted Odds ratio of the SSI rate was 36% lower in 2011 compared to 2008.

**Conclusion:**

The implementation of the bundle was associated with improved compliance over time and a 36% reduction of the SSI rate after adjustment for confounders. This makes the bundle an important tool to improve patient safety.

## Introduction

Surgical site infections (SSI) are frequent and serious complications of surgical procedures. They are associated with a prolonged duration of hospitalization, readmissions, re-interventions and the patient may suffer from permanent disability or even death. [1] In 2007 a study in The Netherlands was performed to quantify the amount of preventable complications and mortality in Dutch hospitals. [2] This resulted in 10 highly preventable complications, with SSI as on of the most important complications. Subsequently the Dutch hospital patient safety program (DHPSP) was developed. The DHPSP supports the Dutch hospitals by knowledge exchange, specific preventive programs and networking opportunities. It started in 2009 and the objective was to reduce the occurrence of preventable deaths with 50% by the end of 2012. One of the programs of the DHPSP is prevention of SSI (http://www.vmszorg.nl/10-Themas/POWI). This program defines a bundle consisting of 4 process measures that should be implemented with a compliance of at least 90%. At the same time the SSI rate is measured to quantify the effect of the interventions on the outcome. The elements of the bundle are perioperative antibiotic prophylaxis, hair removal before surgery, perioperative normothermia and discipline in the operating room. The first three measures are considered evidence based for the prevention of SSI and are included in the current national guidelines (http://www.wip.nl/free_content/Richtlijnen/100720powi%20def.pdf). Discipline in the operating room is considered important but difficult to measure and not very well studied. The DHPSP decided to use the number of door openings during the surgical procedure as a surrogate marker for discipline.

Our objective was to implement the bundle of care in colorectal surgery and measure the effect on the SSI rate while adjusting for confounders.

## Methods

The Amphia Hospital is a large teaching hospital with approximately 45,000 admissions per year, excluding day care. A prospective surveillance for SSI was performed based on the criteria of the Centers for Disease Control. [3] All patients who underwent colorectal procedures from January 1, 2008 until January 1, 2012 were included. Dedicated and specifically trained infection control personnel performed the surveillance. Post-discharge surveillance until 30 days after the procedure was routinely performed.

The following characteristics were recorded: age, gender, ASA-score, length, weight, body mass index, wound class, type of procedure, laparoscopic versus open, elective versus urgent, temperature at the end of surgery, duration of surgery, surgeon, number of colorectal procedures per surgeon during the study period, admission date, date of surgery, discharge date, readmission within the post-discharge period, development of SSI, and mortality within 6 months after the initial procedure. This study was approved by the hospital’s Infection control committee and the board of directors as part of the patient safety programme. The medical ethical committee of the Amphia Hospital in Breda, The Netherlands, waived informed consent for this project.

The bundle as defined by the DHPSP was implemented in 2009. Starting in June 2009 bundle adherence was measured every three months using a random sample of 10 procedures. Normothermia was defined as a temperature between 36.0°C and 38.0°C at the end of the surgical procedure. Perioperative prophylaxis was considered correct when the correct drug was given between 15 and 60 minutes before the incision. Hair removal was preferably not performed and when it was done a clipper had to be used. Use of a razor blade was not allowed. Finally, the number of door-openings was measured from opening of the sterile equipment until the surgical wound was closed. This was done by visual inspection of infection control personal. Besides the number of door openings also the reason was recorded. The target for door movements was <10 per hour. These data were used to feedback and development of strategies for improvement.

The bundle compliance was discussed after each measurement in a multidisciplinary team consisting of surgeons, anesthetists, the head of the operating room, operating room personnel and infection control personnel. A newsletter was distributed among all personnel involved in the surgical process every three months. This included the results from the bundle compliance measurements and recommendations for improvement. The program was supported by the management of the hospital, who also allocated one full time equivalent infection control nurse to the program for surveillance of SSI, bundle measurements and feedback.

The following improvements were implemented during the program:

1. Razors were removed from the hospital and replaced by clippers during 2009 and the first half of 2010.
2. An explicit and uniform protocol for perioperative prophylaxis that could be handled by the anesthesia personnel was implemented during 2009. Before the operation started a time-out procedure was in place, which included the administration of antibiotic prophylaxis.
3. The temperature of the patient was measured during the entire process from the ward to the operating theatre and back to the ward. Based on the findings an isolation blanket was administered to patients on the ward before they were transported to the operating room. Previously this blanket had been administered in the operating theatre.
4. Door openings were subjected to a root-cause analysis. The multidisciplinary team critically assessed the determinants of openings and recommendations for improvement were made. The management of the OR was responsible for implementation of these recommendations. The main interventions were: reducing changes of the team for coffee breaks, making sure all equipment was present before the surgical procedure started and not entering the operating room for social talks during the surgical procedure.
5. For the implementation of the bundle a safety culture was promoted, including correcting each other when bundle adherence was at stake.
6. A newsletter as described before provided feedback after each bundle measurement.

All variables were tested univariate using Fishers exact test or Students T-test. Variables with a p-value <0,2 in univariate analysis were included in logistic regression analysis. 2008 was considered the pre-intervention period. Mortality was compared using Kaplan Meier survival analysis.

## Results

**Figure 1:**
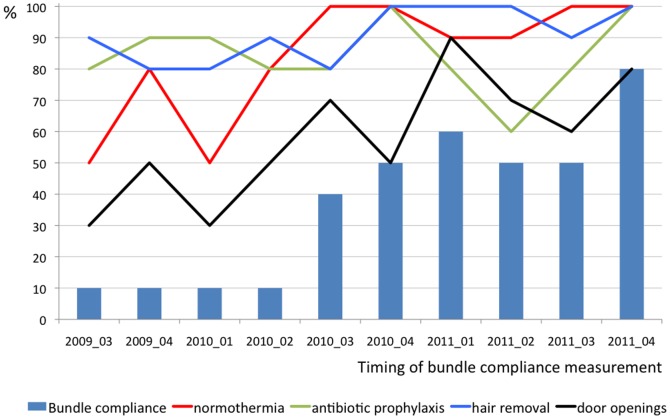
Compliance with the bundle and its individual components in repeated measurements from June 2009 through October 2011.

**Figure 2:**
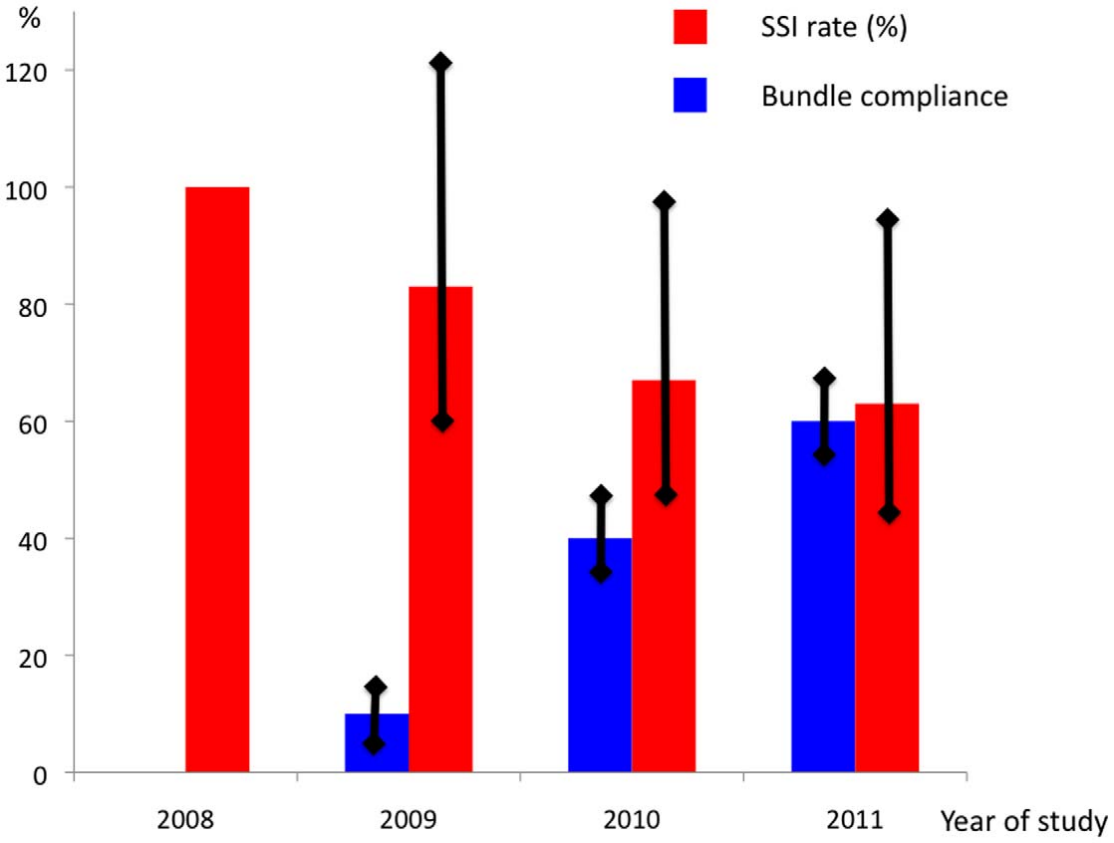
Annual changes in the surgical site infection (SSI) rate and bundle compliance and the 95% confidence interval. Footnote: 2008 was taken as the reference year for SSI and the relative changes after adjustment for confounding variables are provided.

Bundle compliance was measured from June 2009 through October 2011 and increased from 10% to 80% as shown in Figure 1. Also the compliance with the individual components of the bundle are presented. Antibiotic prophylaxis had a relative high compliance during the entire study period. Normothermia and hair removal improved during the process and the compliance was high from June 2010 onwards. Door movements had the lowest compliance overall and never reached a 100% compliance rate during the study. It increased from 30% initially to 80% at the last measurement. In figure 2 the average bundle compliance per year is shown with the 95% confidence interval. Bundle compliance increased significantly from 2009 to 2010 (p<0.001) and from 2010 to 2011 (p = 0.001).

1537 colorectal procedures were performed during the study period and 300 SSI (19.5%) occurred. There were 124 (8.1%) superficial SSI and 176 (11.5%) deep SSI. Table 1 shows the categorical variables in relation to the occurrence of SSI and the statistical significance. The SSI rate was significantly higher in open versus laparoscopic procedures, for surgeons with a lower amount of colorectal procedures, in patients with a higher ASA score or wound class and in non-elective procedures. The SSI rate decreased over time and reached borderline statistical significance. In table 2 the continuous variables are shown. Patients who developed a SSI had a significantly longer duration of the surgical procedure. Also a longer duration of hospital stay was found in patients with SSI (mean additional length of stay: 18 days).

**Table 1:** Categorical variables in relation to the occurrence of surgical site infections (SSI) after colorectal surgery.

Determinant		SSI/N	%	RR	95% CI	p-value
Gender	Female	140/733	19.1			
	Male	160/804	19.9	1.04	0.85–1.28	0.700
Procedure	open	242/1092	22.2			
	laparoscopic	58/445	13.0	0.59	0.45–0.77	<0.001
Number of procedures per surgeon	1–100	162/668	24.3			
	>100	138/869	15.9	0.65	0.53–0.80	<0.001
ASA class	1 of 2	161/959	16.8			
	3,4 of 5	138/559	24.7	1.47	1.22–1.79	<0.001
Wound score	1 of 2	230/1307	17.6			
	3 of 4	70/230	30.4	1.72	1.37–2.17	<0.001
Urgency of procedure	Elective	272/1452	18.7			
	Non-elective	28/85	32.9	1.75	1.28–2.44	0.003
Year	2008	85/394	21.6			
	2009	80/367	21.8	1.01	0.77–1.31	1.000
	2010	74/399	18.5	0.86	0.65–1.14	0.289
	2011	61/377	16.2	0.75	0.56–1.01	0,066

ASA class: American Society of Anesthesiologists classification.

Wound class: Classification based on the intrinsic contamination of the incision site.

**Table 2:** Continuous variables in relation to the occurrence of surgical site infections (SSI) after colorectal surgery.

	With SSI		Without SSI		
	mean	SD	Mean	SD	p-value
Age in years	68.8	11.8	67.4	12.8	0.075
Duration of surgeryin minutes	120.1	57.1	112.1	55.1	0.025
Body mass index in kg/m^2^	25.9	4.6	25.5	4.1	0.189
Length of hospital stayafter surgery	9.7	8.3	27.7	21.1	<0.001

A logistic regression was performed to adjust for confounding as shown in table 3. Most variables that were identified in the univariate analysis retained their statistical significance with the exception of elective versus non-elective procedures. In addition a significant reduction of the SSI rate was observed in 2010 and 2011, with a 36% reduction in the last year of the study, compared to 2008. In figure 2 the reduction of the SSI-rate over time after adjustment for confounders is represented together with the average annual bundle compliance rate. The increased compliance with the bundle was associated with a decrease of the SSI rate.

**Table 3:** Multivariate analysis of variables in relation to the occurrence of surgical site infections (SSI) after colorectal surgery with adjusted Odds ratio’s (AOR) and the 95% confidence interval (CI).

Variable	AOR	95% CI	p-value
Laparoscopic versus open procedure	0.56	0.39–0.80	0.001
ASA class (3,4 and 5 versus 1 and 2)	1.55	1.15–2.08	0.004
Wound score (3 and 4 versus 1 and 2)	1.92	1.33–2.77	<0,001
Number of procedures per surgeon (≤100 versus >100)	1.52	1.14–2.04	0.005
Non-elective versus elective procedures	1.22	0.69–2.17	0.489
Duration of surgery (minuts)	1.006	1.003–1.008	<0,001
age (years)	1.009	0.997–1.021	0.128
Body mass index (kg/m^2^)	1.011	0.979–1.043	0.510
year (2009 versus 2008)	0.83	0.57–1.22	0.345
year (2010 versus 2008)	0.67	0.46–0.98	0.039
year (2011 versus 2008)	0.64	0.44–0.95	0.025

**Figure 3:**
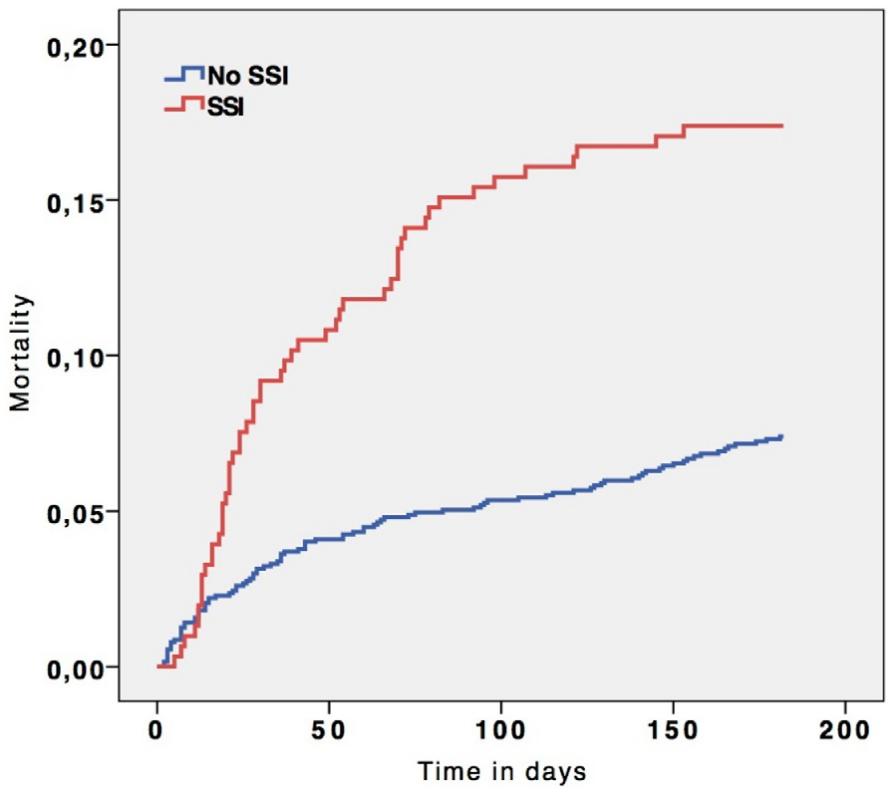
Kaplan meier curve of 6 months mortality in patients with and without a surgical site infection (SSI).

Figure 3 shows a Kaplan Meier curve for 6 months mortality of patients with and without a SSI. A statistical significant difference was found (P<0.001 using the Log rank test). Logistic regression analysis showed that patients with a SSI had a higher likelihood to die within 6 months than those who did not develop a SSI (Adjusted OR: 2.71, 95% confidence interval 1.76–4.18).

## Discussion

The implementation of a bundle of care in our hospital was associated with a substantial (36%) and significant decrease of the SSI rate after adjustment for confounders The relevance of this improvement is obvious, considering the serious consequences associated with SSI. In our study a prolonged length of hospital stay (mean additional length of stay after surgery 18 days) and mortality was found. Moreover, the bundle did not require expensive or complicated interventions. Due to the design of the study we cannot entirely be sure that there have been other unknown factors that contributed to the reduction of the SSI-rate.

A bundle of care consists of a limited number (3–5) of evidence-based recommendations that should be performed during medical procedures carrying a high intrinsic risk of a complication. They are considered important tools to improve the process of care and thereby the outcome for the patient. A zero-tolerance policy is essential, so all bundle components are adhered to in every single patient. In this way the bundle creates a culture of safety.

Bundles for infection control in hospitals have become important since a large multicenter study in the United States of America on intensive care units was performed. The bundle was targeted at the prevention of catheter-related infections. [4] A 66% reduction of the infection rate was obtained. Another successful application was the prevention of ventilator-associated pneumonia. [5] Few bundles for the prevention of SSI have been described to date. An Australian study found a 50% reduction of SSI after colorectal surgery that was not statistically significant. [6] However, the implementation of the bundle was suboptimal and the power was insufficient to draw clear conclusions. Another study used a non-blinded randomized controlled design and found a higher infection rate in the group that was treated according to the bundle. [7] However, this study selected bundle elements that involved technical aspects only which will not affect the safety culture. In addition it is impossible to use a randomized controlled study when a change in the behavior is part of the intervention. Health care workers cannot change behavior based on an individual randomization scheme. In contrast changing the culture is often an arduous process that takes a long time to achieve. In our case the door openings clearly improved after 2,5 years of implementation but never reached 100% compliance.

The bundle that we used was developed by the DHPSP and it effects have been described before. [8] This study involved 284 surgical procedures and studied the relation between de development of an SSI and the application of the bundle and its components in individual patients. In this relatively small study a significant relation was found between the development of a SSI and higher number of door openings. The authors did not explore the reason for the higher number door openings and therefore, could not exclude that there were confounding factors that caused both the higher number of door openings and the development of a SSI, e.g. complications during surgery. To our knowledge there are no other reports describing the number of door openings in relation to the development of SSI. However, a recent study found a strong correlation between noise levels during the surgical procedure and the development of SSI. [9] Also, talking about non-surgery-related topics was associated with a significantly higher sound level. The authors conclude that intraoperative noise volume was associated with SSI and that this may be due to a lack of concentration, or a stressful environment, and may therefore represent a surrogate parameter by which to assess the behavior of a surgical team. The door openings in our study likely reflect the same factors, at least in part. During our visual inspections it was observed that social talk and replacement of the team for coffee breaks were important factors that increased the number of door openings. Also the lack of having all necessary equipment ready before the procedure starts indicates a suboptimal process which may create distraction and stress during the procedure, once the equipment is needed. Changing this behavioral aspect was the major challenge of this project. It required a change of the daily procedures, which took many discussions and repeated feedback. Although major improvements have been made we consider this part not fully implemented at the end of the study. In 2012, the management of the operating theatre introduced a system of “yellow and red cards”. A yellow card initially warned personnel who did not adhere to the agreed procedures. This meant that repeated failure to comply would be followed with a temporary suspension from the operating theatre (red card). This reflects the zero-tolerance approach that is needed for optimal implementation of this kind of bundles. We probably should have implemented this in an earlier phase of our project to achieve a more rapid compliance with the bundle. Nevertheless, significant improvements were obtained from 2009 to 2010 and again in 2011. Major improvements for bundle adherence were initially realized for preoperative hair removal and for peri-operative normothermia. These interventions were relatively easy and cheap. The isolation blanket was already provided to patients but only when they arrived at the operating room. Analysis of the process showed that patients cooled down on average 1.0°C when they left the ward until they arrived in the operating complex. The active warming that was applied during surgery using a Bair Hugger system (Arizant Healtcare inc, Minneapolis, USA) started with a significant loss. Simply administering the isolation blanket on the ward before the patients left improved the core temperature of the patient after surgery. The hair removal required removal of razor blades and the introduction of clippers on all wards (Several wards already used clippers before the start of the program).

The costs of the project were mainly the infection control practionner who performed the surveillance and bundle measurements. The annual costs can be estimated at €40.000 per year. The benefits are likely much higher. A simple estimate can be made by assuming that the program reduces the SSI rate that was observed in 2008 (21.6%) by approximately 35%. On average approximately 375 colorectal procedures are performed each year. The annual number of SSI prevented would be 21. Each of them is associated with 18 days of additional length of stay (annual total: 378 days at €500 per day) and a 2.7 higher chance of mortality. This would mean that each year 3 deaths would be prevented. This extrapolation is not entirely valid since patients that develop an SSI may have underlying reasons that cause a longer length of stay or higher risk for mortality also when they do not develop an SSI. However, a recent randomized controlled study that evaluated a specific intervention to reduce the SSI rate also found that this was associated with a reduction of the length of stay and mortality. [10] It is therefore likely that the reduced SSI rate is associated with a cost saving that is higher than the investments.

With this study we show that the bundle as defined by the DHPSP can be implemented. We have used a quasi-experimental design with correction for confounding variables to determine the effect on the SSI-rate. We did not perform an interrupted time series analysis since the interventions were not implemented within limited time periods but during periods of approximately one year (normothermia and hair removal) or even during the entire study period (door openings). The improvements with implementation of the bundle were followed by subsequent reductions in the SSI rate. The process was prolonged with frequent feedback and discussions that will also have caused other changes in behavior that were not included in the bundle as it was defined. These unknown factors may have contributed to the reduced infection rate as well. Also in the year before the bundle was implemented our hospital participated in the SURPASS study that introduced a time out procedure and a preoperative checklist. [11] Although this study had been finished before we started the current project we cannot exclude that there was a residual effect of the checklist during the study period. Other study designs that control for confounding and for the Hawthorne effect are preferred but cannot be applied when a culture change is part of the intervention as discussed above. We therefore consider a quasi-experimental design, including adjustment for confounding variables, as the optimal method to study the effects of this intervention.

The results of the DHPSP are measured in a national surveillance program called PREZIES (www.prezies.nl). In 2009 the average bundle compliance in Dutch hospitals was 5%, it increased to 11% in 2010 and 10% in 2011 (personal communication). Others [8] and we have shown that a better compliance is achievable and this was associated with a substantial and significant reduction of the infection rate. Therefore, we found this bundle a useful tool to achieve a culture of safety in the operating theatre and thereby probably improving patient safety for surgical patients by reducing the SSI-rate.
